# Proposing a minimal set of metrics and methods to predict probabilities of amyloidosis disease and onset age in individuals

**DOI:** 10.18632/aging.202208

**Published:** 2020-11-18

**Authors:** Richard S. Criddle, Hsien-Jung L. Lin, Isabella James, Ji Sun Park, Lee D. Hansen, John C. Price

**Affiliations:** 1 Department of Chemistry and Biochemistry, Brigham Young University, Provo, UT 84602, USA

**Keywords:** amyloidosis, protein folding, transthyretin, proteostasis

## Abstract

Many amyloid-driven pathologies have both genetic and stochastic components where assessing risk of disease development requires a multifactorial assessment where many of the variables are poorly understood. Risk of transthyretin-mediated amyloidosis is enhanced by age and mutation of the transthyretin (TTR) gene, but amyloidosis is not directly initiated by mutated TTR proteins. Nearly all of the 150+ known mutations increase dissociation of the homotetrameric protein structure and increase the probability of an individual developing a TTR amyloid disease late in life. TTR amyloidosis is caused by dissociated monomers that are destabilized and refold into an amyloidogenic form. Therefore, monomer concentration, monomer proteolysis rate, and structural stability are key variables that may determine the rate of development of amyloidosis. Here we develop a unifying biophysical model that quantifies the relationships among these variables in plasma and suggest the probability of an individual developing a TTR amyloid disease can be estimated. This may allow quantification of risk for amyloidosis and provide the information necessary for development of methods for early diagnosis and prevention. Given the similar observation of genetic and sporadic amyloidoses for other diseases, this model and the measurements to assess risk may be applicable to more proteins than just TTR.

## INTRODUCTION

The goal of this article is to describe how a minimal biophysical model of amyloidosis can guide diagnosis and treatment of relevant diseases. Towards that end we first go over the literature support for the model. We then describe current experimental methods to make the necessary measurements. Finally, we discuss the way application of this model could change our understanding of the etiology and treatment of TTR amyloidosis specifically. Earlier reviews [1–5] provide extensive coverage of the fundamental information available from laboratory and clinical work on amyloidosis. Therefore, brief summaries of relevant information on the TTR protein properties and TTR amyloid diseases are given here with only limited further references.

### Transthyretin (TTR) protein amyloid

**Figure 1:**
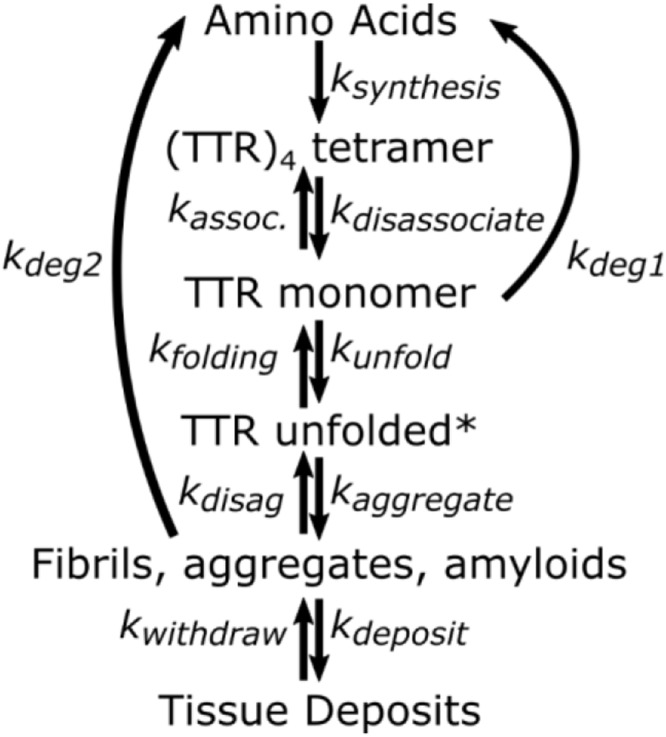
**Minimal kinetic mechanism for TTR homeostasis.** TTR unfolded* is a transient intermediate that may not accumulate to a measurable degree. It is assumed that degradation of fibrils and aggregate is negligible compared to degradation of monomer (k_Deg2_<<<k_Deg1_).

Human transthyretin (TTR, Uniprot # P02766) is a tetrameric, beta-sheet-rich, blood protein important in the transport of thyroxine and retinol. Blood-borne TTR is produced and secreted from the liver predominantly, although the choroid plexus of the brain does express high amounts [6]. In plasma, the TTR tetramer is in dynamic equilibrium with a monomeric form (Figure 1) with the degree of dissociation dependent on individual genetics and exogenous factors like binding of small hormones. TTR is less stable in monomeric form. TTR monomers may misfold and be incorporated into supramolecular arrangements of beta-sheets known as cross-beta structures [7–10]. These cross-β assemblies are stabilized by an extensive network of hydrogen bonds that cause them to form insoluble amyloids and resist proteolysis. Initially, misfolding is a spontaneous process [11, 12]. Once present, TTR oligomers may act in prion-like fashion [13, 14] to catalyze further misfolding of TTR into cross-β assemblies that accumulate into the fibrils and larger TTR amyloids as observed with multiple other amyloidoses [7, 15].

### Biological factors promoting amyloidosis

Three general factors have been linked with certainty to the development of TTR amyloidosis: age, gender, and stability of the TTR tetramer. Development of TTR amyloidosis increases with age, occurs more frequently and earlier in men than women, and is enhanced by mutations that decrease structural stability of TTR tetramers [1–5]. It is unknown how these factors impact the half-life in plasma. The removal pathways have not been fully characterized, but in rats occurs predominantly in the liver [16]. TTR can be removed from plasma by proteolysis of dissociated monomers, and and by aggregation into fibrils, β–amyloids, and amyloid deposits in tissues. The rates and mechanisms for removal of fibrils and amyloid deposits are poorly understood, if indeed such pathways are significant. The relative amounts of TTR removed via these pathways depend on the relative rates of two processes, proteolysis and amyloid formation. Both are preceded by tetramer dissociation to monomers (Figure 1). If proteolysis is rapid enough to maintain TTR monomer concentration sufficiently low, transformations leading to amyloid deposits are not significant. With rapid protease activity, TTR monomer concentration in plasma is determined by tetramer stability. But when protease activity decreases relative to synthesis, monomer accumulates and can aggregate. Aggregation has an n^th^ order dependence on monomer concentration and even a small increase of monomer concentration greatly increases the probability of oligomer formation [14].

Amyloid accumulation can be slowed by small molecules which bind and stabilize the tetramer (tafamidis, diflunisal, and AG10), and limit monomer formation [17, 18] and thus slow but do not cure the disease. Another treatment approach arises from the observation that the TTR plasma concentration in an individual is maintained at a near constant value both before and after an amyloidosis develops [19, 20]. The rate of removal due to protein degradation generally decreases with age [21–23]. It is expected that if proteolysis slows to where it cannot keep up with the rate of formation of monomers, TTR is removed by formation of fibrils and β-amyloid deposits. We therefore hypothesize that the age-related imbalance in proteolysis initiates TTR amyloid diseases. Identifying the proteolysis pathways would provide the next step toward developing a preventive cure for TTR amyloidosis.

### Individual differences in development of amyloidosis

Differences in lifestyle factors like diet, internal physiology, and mutant variants contribute to individual differences in the timeline of amyloidosis development. All three factors quantitatively affect the dissociation of the tetramer into monomer [9, 24] and because removal presumably proceeds via monomer, the relative rates of proteolysis and aggregation differ among individuals. Any TTR mutation, physiological condition, or exogenous factor that stabilizes the TTR tetramer also decreases monomer concentration. This favors a smaller ratio of aggregation rate to proteolysis rate, which delays or prevents amyloidosis. Based on clinical reports we expect physiological differences such as differences in plasma proteins and small binding molecules affect tetramer stability and thus development of amyloidosis [1, 3, 25–29]. These physiological differences may explain differences in the age of onset in men and women, but the *in vivo* studies required to quantitate these effects have not yet been done. Exogenous factors related to life-style and diet which modify metabolites and hormone signaling may also be important modulators of amyloidosis, but the significance of these factors is also unknown.

Mutations in the gene coding for TTR were evolutionarily selected to enhance its role as a transporter of thyroxine and retinol, but play an important role in amyloidosis. Since TTR mutations affecting amyloidosis become evident after the age of reproduction, no selective Darwinian pressure hindered acceptance and propagation of mutations that affect amyloidosis as long as the mutations had no substantial effect on transport functions. As a consequence, the TTR gene exhibits a large number (150 and counting) of antagonistic variants and a few protagonistic variants such as T119M, V30M, and L55P. [30, 31]. The most common variant is designated as ‘wildtype’.

The many mutations of TTR proteins appear to have minor effect on the ability of the monomers to be incorporated into β-fibrils and amyloids [9, 14]. All form remarkably similar wrapped, lamellar cross β-sheet structures [14, 31]. Even though mutant changes in single amino acids at the various locations on the TTR protein do not appreciably interfere with formation of β-sheet aggregates, these same changes do cause different tissue tropisms and corresponding differences in phenotypic expressions of the disease according to genotype [32]. For example, the amyloids formed from wildtype TTR show a predominantly cardiac tissue tropism where the β-amyloids accumulate in heart muscle. But, the symptoms often differ among individuals having the same TTR mutation and may range from carpal tunnel syndrome to neuropathy, spinal stenosis, gastrointestinal problems, Hashimoto’s disease, etc. and various combinations of these.

Some of the diversity in symptoms may arise because mutant subjects are frequently heterozygous with wildtype and mutant TTR co-expressed in equal amounts. Tetramer formation from mutants produces mixed hetero/homo-tetramers (i.e. wt_4_; wt_3_mt_1_; wt_2_mt_2_; wt_1_mt_3_; mt_4_). When the tetramers dissociate and monomers aggregate into fibrils, both wildtype and mutant subunits can be incorporated into the β-sheet structured oligomers. The fibrils thus contain mutant-specific variations in the protein structures at their surfaces that may create reactive surfaces which bind to cellular membranes with complementary binding groups. Mutant-specific differences in oligomer/fibril binding to cellular membranes could explain differing phenotypic patterns of TTR amyloidosis.

### An integrated model for transthyretin (TTR) amyloidosis.

Studies of dissociation of tetrameric TTR have implicated both kinetics and thermodynamics in the amyloidogenic behavior of TTR. Two observations in the literature suggest that focusing on kinetics may be important: 1) the concentration of monomer in equilibrium with the tetramer is orders of magnitude lower than the concentration of tetramer [9, 26, 33–35], and 2) the exchange rate of monomers between wild-type tetramers is much faster at 4° C than at 25° C and even slower at 37° C [36]. Taken together, these two observations indicate the tetramer undergoes a fast dissociation followed by a temperature-dependent conformational change of the monomer. As a consequence of the conformation change in the monomer, at 4° C the association is much slower than the dissociation reaction and allows promiscuous mixing of monomers. This model accounts for both the slow exchange rate and inverted temperature dependence.

Figure 1 graphically summarizes the relation of amyloidosis to TTR concentrations, stabilities of TTR tetramers ((TTR)_4_), and fates of monomers (TTR). This graphic makes it clear that quantifying the relative rates for monomer transformations is key to understanding development or lack of development of amyloidosis. The first step in removal of TTR is dissociation of the tetramer to monomer. Monomers that are not removed by proteolysis irreversibly aggregate into fibrils and β-amyloid deposits. The path from monomer to β-amyloid deposits occurs extracellularly and may include misfolded monomers, protofilaments, filaments, and fibrils, but it is generally agreed that monomers are folded into β-structures before, or upon their incorporation into, fibrils and maintain this structure in the fibrils and further into the construction of amyloids [37].

Formalizing a minimal kinetics mechanism (Figure 1) allows us to build a quantifiable model. Assuming the rate of dissociation and association between monomer and tetramer is fast relative to the rates of proteolysis and aggregation, the rates of formation and removal of TTR from plasma are described by the following equations.

$\text{Amino}\;\text{acids}\to {\left(\text{TTR}\right)}_{4}$

$$\text{d}[{\left(\text{TTR}\right)}_{4}]\text{/dt}=+{\text{k}}_{\text{synthesis}}$$ (1)

TTR→amino acids

$$\text{d}[\text{TTR}]\text{/dt}=-{\text{k}}_{\text{Deg1}}[\text{TTR}]$$ (2)

TTR→growth of TTR aggregates

$$\text{d}[\text{TTR}]\text{/dt}=-{\text{k}}_{\text{aggregate}}[\text{TTR}]$$ (3)

Assuming the rate of production of TTR tetramer (equation 1) is constant, proteostasis is maintained by balancing the rates of removal (equations 2 and 3) with the rate of production, and therefore the sum of the rates of reactions 2 and 3 must equal 4*k_synthesis_ (equation 4).

$${\text{k}}_{\text{Deg1}}[\text{TTR}]+{\text{k}}_{\text{aggregate}}[\text{TTR}]=4({\text{k}}_{\text{synthesis}})$$ (4)

Solving for the concentration of monomer, [TTR] gives

$$\left[\text{TTR}]=\text{4}({\text{k}}_{\text{synthesis}})\text{/}({\text{k}}_{\text{Deg1}}+{\text{k}}_{\text{aggregate}}\right)$$ (5)

Equation 5 suggests the concentration of TTR monomer depends on the relative values of the rate constants for the production and removal processes. The removal rate constants change with conditions that affect the rates of removal of the monomer. In the absence of aggregate formation, equation 5 predicts that the plasma monomer concentration is a constant that depends solely on the stability of the tetramer. Also, the ratio of k_synth_ to k_Deg1_ may differ between individuals, so the concentration of monomer may differ as well. Therefore, an individual’s TTR amyloidosis risk could be assessed by measuring the individual’s serum TTR concentration and the stability of their TTR tetramer with respect to the monomer. The greater the concentration of tetramer and the lower the stability, the greater the chance that amyloid deposits will cause disease earlier in life. The ratio of a quantitative measure of tetramer stability to TTR serum concentration over time in a single individual (longitudinal) or across populations (cross-sectionally) at specific ages is thus predicted to be a useful index of the likelihood of an individual developing a TTR amyloid disease later in life. Individuals with the most destabilizing mutations or cellular conditions in which amyloidosis is initiated at earlier ages would need to be compared as a unique population cross section or longitudinally.

**Figure 2:**
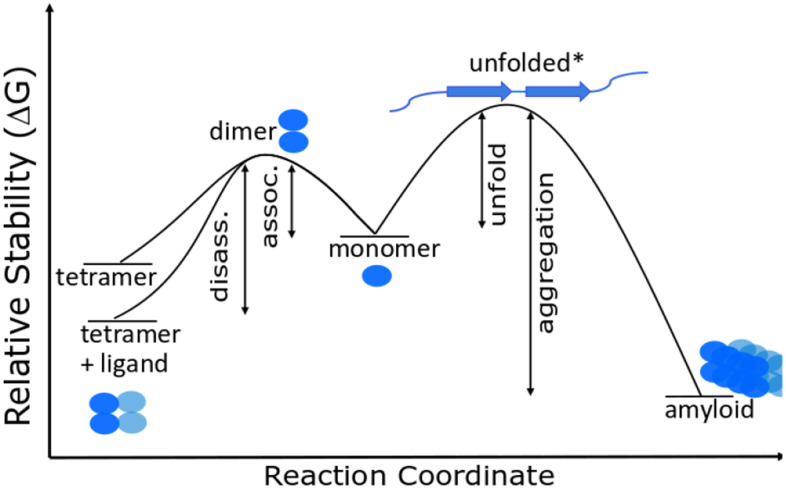
**Gibbs energy diagram for dissociation/association reactions of TTR.**

These relative stabilities in combination with the frequently observed fact that unfolding of protein tertiary and secondary structures are endothermic [38–40] allows us to construct a Gibbs energy diagram for amyloidosis (Figure 2).

$${\left(\text{TTR}\right)}_{\text{4}}↔\text{4TTR}↔{\text{4TTR}}^{\text{mis}}$$ (6)

Here TTR^mis^ is the misfolded monomer, either unfolded or a β-pleated sheet structure. Figure 2 shows that any change that decreases the Gibbs energy of the tetramer increases the activation energy for dissociation and decreases the concentration of monomer whether the monomer concentration is determined by thermodynamics or by kinetics. Drugs that bind to and stabilize the tetramer therefore decrease the monomer concentration. The figure also illustrates that misfolded monomers *in vivo* are tagged and hydrolyzed or aggregated because refolding and reentering the tetramer structure has a very low probability, i.e. amyloids are very stable relative to the unfolded intermediate. The likelihood of developing an amyloid disease is thus predictable from the Gibbs energy for dissociation of the tetramer and the serum concentration of an individual. Relative Gibbs energies of mutant variants and the effects of drug binding can be measured in near *in vivo* conditions as shown below.

### Review of methods for determining TTR tetramer concentration and stability in serum samples from individuals

Measuring an individual’s TTR concentration and stability within the context of their blood serum is important clinically because it accounts for individual variations in environmental variables like hormone concentration or TTR binding partners. Any variable which may change TTR conformational distributions and stabilities would change the predicted risk of amyloidosis.

Although multiple methods exist to measure concentration and stability, we discuss methods which seem to us most relevant and easiest to employ. Current mass spectrometry based methods represent a viable approach for monitoring both concentration and structural stability within the complex mixture of the blood. These methods are simple to multiplex and currently employed for a wide variety of clinical diagnostics [41–43].

### Concentration measurements

**Figure 3:**
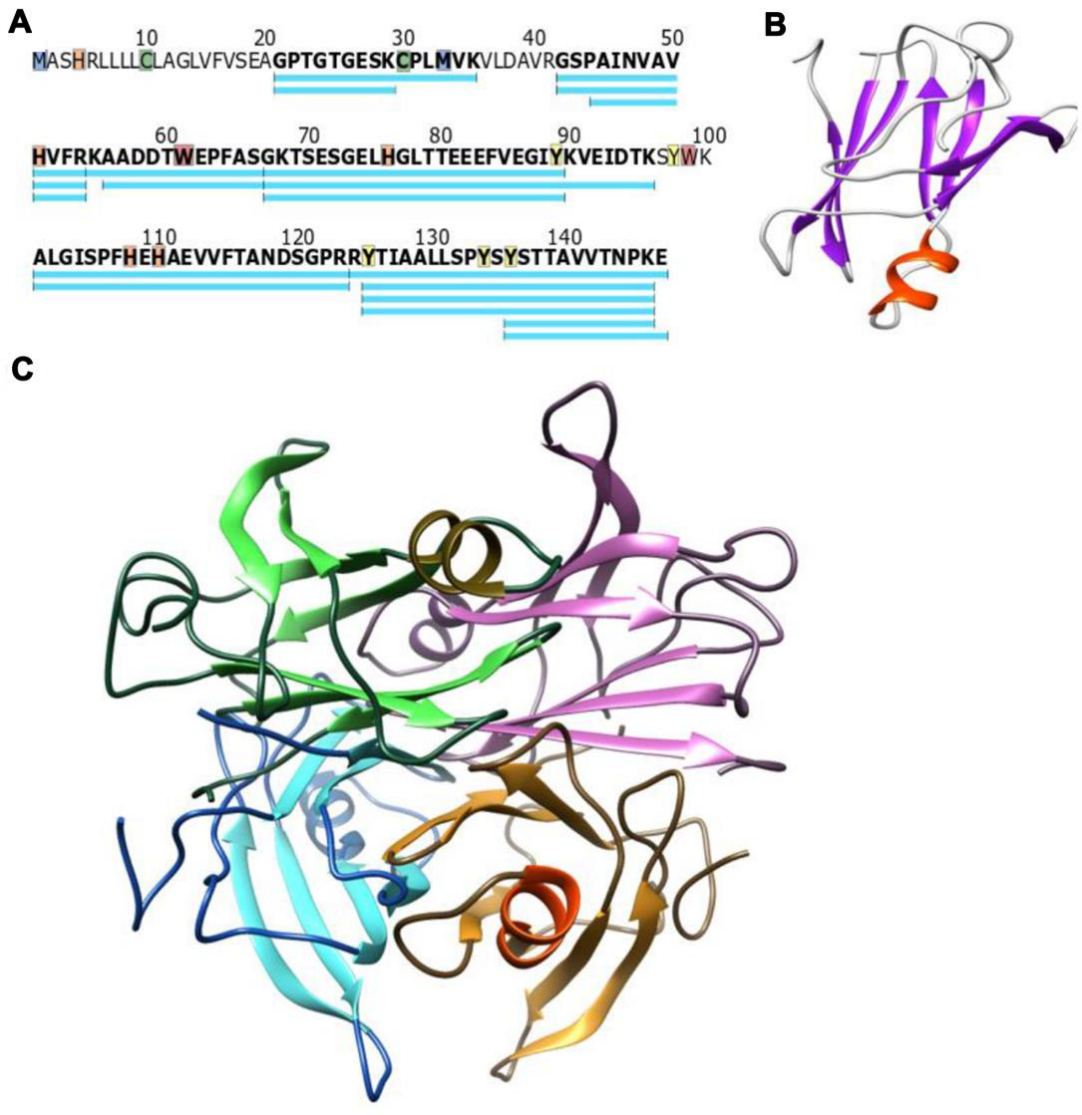
(**A**) Canonical WT TTR sequence, peptides frequently observed in mass spectrometry experiments are shown in blue. Highlighted residues have published chemical modifications that could serve as fold-stability markers (**B**) The TTR monomer folds into two discrete beta sheets and a small alpha helix (PDB structure 1BZE) (**C**) The consensus model of the tetramer has four monomers (each in a different color) interacting along the edges of the beta sheets which would stabilize the protein structure in these regions (Model incorporates PDB 1BZE and 1BZ8).

Multiple high quality reviews have been published on the topic of mass spectrometry for measuring protein concentration [44–47]. An optimized clinical assay for TTR might include a panel of internal standards composed of synthetic isotopically labeled peptides versions of natural TTR peptides (Figure 3A). This type of test could allow for simultaneous measurement of TTR concentration and test for known pathogenic mutations. Panels of internal standards for concentration measurement are commonly used for small molecules like drugs of abuse as well as protein specific measurements [48] and optimized assays are highly specific, require very little sample, and can be run on thousands of samples around the clock. Little or no change beyond selection of the peptides to be measured are required to monitor TTR versus any other protein.

### Stability measurements

Protein structural stability has historically been assessed as the resistance to denaturation [49, 50]. As shown in Figure 3 the monomer sequence Figure 3A folds into both beta sheet and alpha helical secondary structures Figure 3B. The relative resistance to denaturation can be estimated based on non-covalent bonding within the secondary structure, and would be different in an alpha helix versus a beta sheet. If a reporter is located in a beta sheet rich region versus the alpha helical region, different stabilities would be expected based on the location of the reporter and the folding state (unfolded, monomeric, tetrameric, or amyloid) of the protein. For example, residues 95 to 101 are within the alpha helix and thought to be distant from any protein-protein interfaces in the tetramer (Figure 3C based on reference [33]). Based on the structure in Figure 3, the alpha helical section of the protein may have a similar structural stability regardless of protein oligomeric structure (tetramer versus monomer). The edges of the beta sheets on the other hand gain new binding interactions in the tetramer and would be significantly modified relative to the monomer. In amyloid, there is an overall loss of alpha helical structure and increase in the beta sheet for each monomer [5]. Thus, measuring the fold stabilities along the entire sequence of the protein could be valuable for differentiating between monomer, tetramer, and fibril/amyloid forms of TTR. Further, measuring the TTR structural stability in the context of blood serum (in situ) is important as ligands within the blood serum bind and stabilize the tetramer beta sandwich which could be read out as a shift in the structural stability of these sections of the protein.

**Figure 4:**
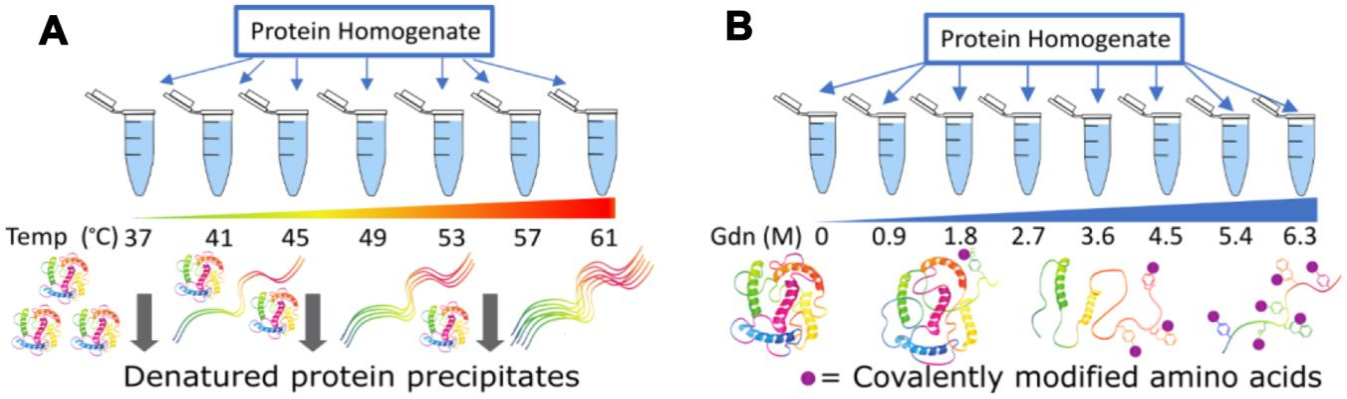
**Two methods to measure protein structure stability via mass spectrometry.** In (**A**) concentration of the remaining soluble protein is measured, while in (**B**) the relative amount of modification for amino acid side chains is the reporter.

Several mass spectrometric techniques have been published recently which allow in situ measurements of structural stability along the entire sequence of the protein [43, 51–53]. These methods can be generalized to chemical versus temperature denaturation of the protein (Figure 4). Both methods can measure sequence specific stabilities across TTR within a complex mixture of proteins like blood serum. Both methods also measure individual unfolding curves within complex mixtures by treating small aliquots to gradually increasing denaturation conditions. Each method has individual advantages and disadvantages. Thermal is fast, requires minimal sample handling, and uses the entire protein sequence as a reporter. However, the thermal mechanism functions through a precipitation step that is not fully characterized and may change with unknown variables. The chemical method uses covalent modification of specific amino acids to report unfolding. This is an advantage in that all the protein stays in solution and both labeled and unlabeled forms of the peptide can be measured and signal compared for substrate and product. The disadvantage of the chemical method is that the distribution of modifiable amino acids is nonuniform across any protein. For example, methionine labeling has been used to monitor chemical denaturation and has the sensitivity to identify stability changes due to drug binding [54] or secondary structure differences [55]. Unfortunately, only a single methionine is found in the N-terminal portion of the TTR protein (Figure 3A). Fortunately, a variety of different labeling methods exist in the literature that may be appropriate for monitoring protein structure [56].

**Figure 5:**
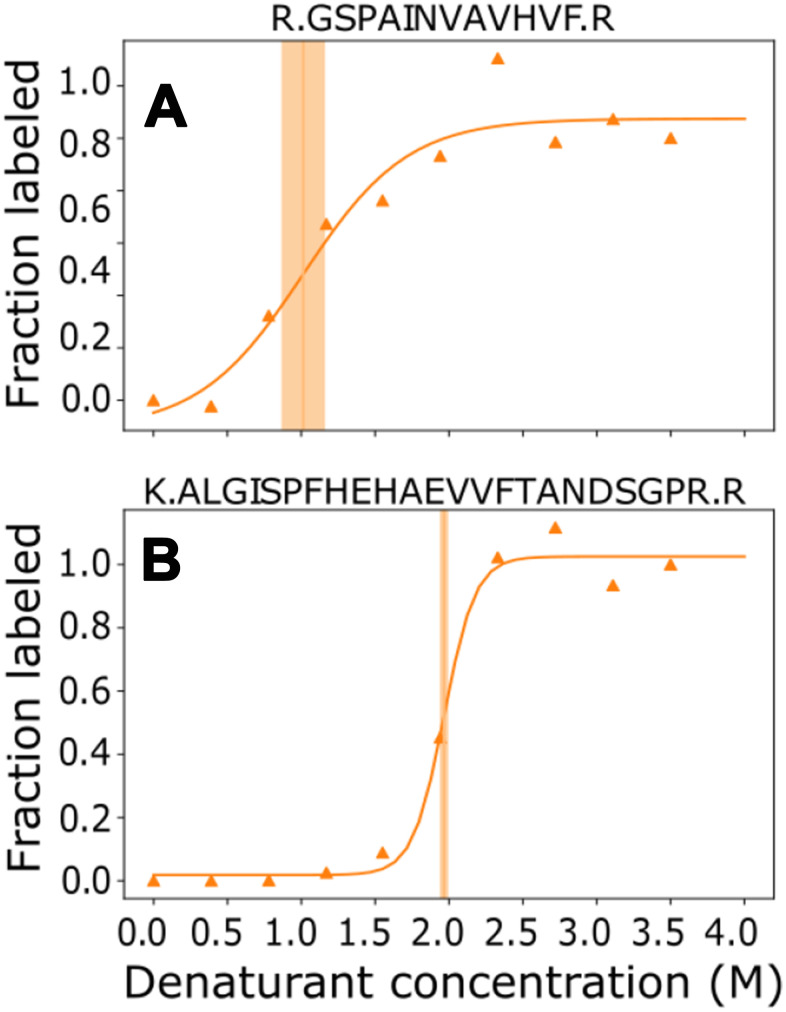
**TTR structure causes modified susceptibility to denaturation for different parts of the sequence.** Individual measurements (triangles) were fit across the denaturant concentrations to calculate the midpoint (vertical line) and confidence interval of the midpoint (shaded area) for two representative peptides (Panel **A**: amino acids 43-55, Panel **B**: amino acids 101-123).

Most of the published studies using mass spectrometry unfolding assays have used yeast or cell culture. To test whether these mass spectrometry methods might be appropriate for blood serum we performed preliminary tests. We found the thermal denaturation technique was not effective for blood serum. We tested temperatures up to 100° C, but there was no evidence of precipitation. This may be due to the extreme thermal stability of Albumin [57], stabilizing the rest of the proteins in the serum. In comparison, proteins from tissue homogenate precipitated efficiently beginning at 40C, and precipitation increased as predicted up to 70C, at which point 90% of the protein had precipitated from solution, as reported in the literature [52]. To test the chemical denaturation method, we used guanidine denaturation and histidine modification by iodine to assay blood serum. We observed differential behavior for several peptides and measured significant differences in the denaturation midpoint of two different sections of TTR (Figure 5). Although both of these sequences (A: 42-53, B:101-123) are expected to contain beta strands, the peptide A has a lower overall stability (denatures at lower concentrations) in these conditions than peptide B.

After optimization of modification chemistry for residues in sections of interest, this type of method has the potential to provide the stability measurements needed to assess risk of amyloidosis. This will require significant effort to validate in a cross-sectional study, and we expect that longitudinal measurements could be most fruitful if made throughout a subject’s lifetime.

## DISCUSSION

There are more than 30 characterized amyloidosis diseases involving a variety of proteins. While these diseases differ markedly in their physiological effects, there are common features in their development. Multiple mutations within the various precursor proteins promote amyloidosis (eg. TTR>150 known mutants, gelsolin>4, lysozyme>10, and fibrinogen>18) [58–61]. Most amyloidogenic mutations change a single amino acid, yet these simple changes give rise to multiple phenotypic disease symptoms that differ between different mutations of the same protein and among individuals with the same mutation. The individual nature of the mutations in each of these diseases presumably plays a direct role in determining differences in the oligomeric β-sheet structures of precursor protein and impacts the phenotypic expression of the diseases [62]. All these diseases have individual variability in age of onset, indicating that the mutation is not the triggering factor for initiation of amyloidosis. The nature of factors initiating onset of amyloidosis is not currently known for any of these diseases, However, the common features suggests that a model developed for TTR amyloidosis may be applied in analysis of the other heritable amyloidosis diseases.

The proposed model emphasizes that the relation between monomer concentration and proteolysis is important to understanding development of amyloidosis. Spontaneous unfolding of monomer is likely variant dependent or can be triggered by a change in the molecular environment [49]. The misfolding of monomer into β-sheet and aggregation varies among individuals based on genetics and lifestyle decisions which modify the concentration of endogenous small molecules which stabilize the TTR tetramer. Therefore, our metrics of relative TTR tetramer stability in the presence and absence of putative stabilizers should be measured *in vivo* or at conditions as close to *in vivo* as possible.

**Figure 6:**
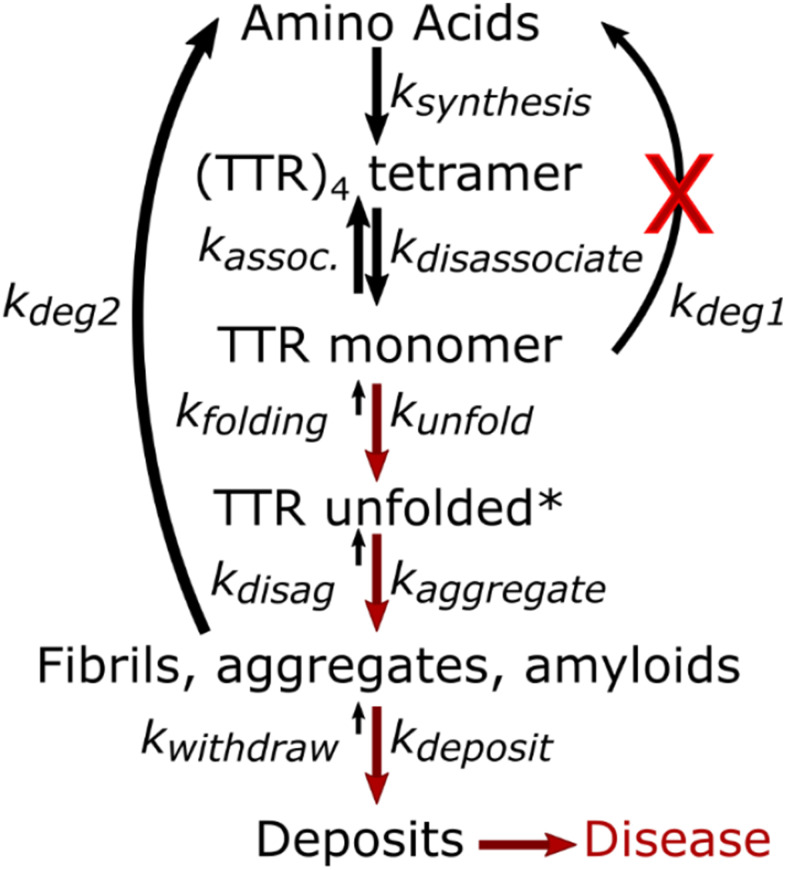
**Age dependent changes leading to amyloidosis disease.**

Proteolysis is generally rapid enough to maintain the monomer at concentrations that minimize aggregation. However, the age dependence for amyloidosis risk could be connected to decreases in protease or chaperone activities that occur at older ages [21–23, 38]. Decreases in protease activity (k_Deg1_, Figure 6) and chaperone activity (k_folding_ and k_disaggradation_) would increase monomer concentration and increase the unfolding rate causing a greater fraction of monomer to aggregate. This could explain why amyloidosis typically occurs later in life. The rate of proteolysis of TTR has a linear dependence on the monomer concentration, but initiation of TTR aggregation has a higher order dependence on monomer concentration, and growth of TTR aggregates likely causes amyloid deposit growth rates to increase exponentially once deposition begins. An exponential increase in rates of oligomer formation to maintain proteostasis in the face of the increase in monomer concentration would explain the rapid progression of disease once symptoms are apparent.

Accumulation of abnormal proteins is a common feature of senescent tissues [63, 64]. This suggests that the decoupling of the synthesis, folding and proteolytic activities is general and related to the protein quality control in cells [21–23]. But, decreased proteolysis and increased misfolding may also be caused by modifications that make the altered proteins less susceptible to proteolysis, e.g. amino acid side-chain modifications, chemical crosslinking and/or aggregation, may result in the altered proteins being poor substrates for the proteases. In cases where specific proteins are chemically tagged for proteolytic turnover to maintain homeostasis, a decreased rate of the tagging reactions could also cause a decreased turnover rate of specific proteins and an increase in monomer concentration.

Support for the contribution of cellular quality control as the initiating variable comes from twin studies. Twins with the same mutation may express symptoms of amyloidosis at different ages [28]. Relative rates of protein turnover vary among individuals, depending on gender, diet, genetic background, etc. According to the model, individuals who maintain adequately high rates of proteolysis of TTR do not get amyloidosis, but an “adequate” proteolysis rate is a function of the stability of the tetramers of various mutants and individual physiology. An age-related decrease in proteolysis rates of individuals with TTR destabilizing mutations increases monomer to the threshold concentration for aggregate formation. This threshold is reached earlier and deposition of amyloids is maintained at higher rates with variants having less stable TTR tetramers in their individual plasma background. This could explain the findings that calorie restriction (which reduces global protein synthesis [65–68]) protects against amyloid deposition [69–71]. Reducing synthesis to match the diminished proteostasis capacity could result in many of the same benefits as therapeutically increasing proteolysis and chaperone activity.

In current practice, TTR amyloidosis has been divided into two amyloid diseases referred to as wild type and familial. Wild type is described as an “age-related, non-hereditary systemic amyloidosis” that is induced by unknown factors to form amyloid deposits by wild type TTR proteins. The familial disease is described as a separate disease that occurs as a consequence of mutations of wild type TTR protein. We disagree with this separation because the wild type and familial diseases share a common mechanism even though manifestations of the disease may differ. The wild type TTR gene underwent Darwinian selection for transporting thyroxine and retinol when reproductive age and life expectancy were much shorter than today and amyloidosis had negligible effect. Any mutations in the TTR gene that did not have a significant adverse effect on transport functions were accepted and propagated resulting in 150+ recognized mutant forms of TTR that generally differ from wild type by a single amino acid. These mutant proteins may enhance, decrease, or have no effect on TTR tetramer stability compared with wild type. All forms of TTR, including wild type, can cause amyloidosis and the propensity for amyloidosis in people with wild type TTR is just as heritable as it is for those with a mutant form. Thus, there is no reason to consider wild type TTR amyloidosis and familial TTR amyloidosis as separate diseases. Separate clinical trials of effectiveness of treatments for wild type TTR and familial TTR amyloidosis are unnecessary and counterproductive. However, our model does not address why various mutants often cause predominantly cardiac, or neurological, or other diseases and trials comparing treatments for differing diseases should be done. Individuals with the same mutant form of TTR commonly exhibit different disease phenotypes because of differences in tissue tropisms. Grouping TTR diseases by tropisms running drug trials with (a) predominantly cardio, (b) peripheral neuro, or (c) a combination of both reduces effort and increases efficiency. Our model and experimental methods reported here offer a solution to this conundrum by rapidly quantitatively examining properties of TTR variants in near *in vivo* conditions to evaluate drug responses in individuals. The same general model and methods may also apply to other late-in-life expressed amyloid diseases. Thus, we propose individual testing for predicting response to treatment instead of clinical trials.

Current treatments for amyloidosis based on altering structural stability will not lead to a cure. A cure requires identifying and correcting the factor(s) that are responsible for the changes in relative rates of monomer removal and amyloid deposition. Our model does not identify these factors but suggests metrics that that future research can use to interrogate the age-related changes in protease activity, chaperones, post translational N-glycation, cellular energetics, oxidative reactions, and glycosylation.

For example, glycosylation has been implicated in TTR amyloidosis [24, 72–74]. Glycosylation is a viable candidate to consider for an initiating event in TTR amyloidosis because (a) it is commonly involved as a post translational signal in biochemical pathways to activate or moderate metabolic pathways [75], (b) glycosylation activity in human plasma is heavily influenced by gender [73], (c) there is a general decrease in glycosylation activity with age [76]. TTR is generally synthesized and secreted from the liver without added sugars, though it has a consensus sequon for glycosylation at Asn 98. Crystallographic data suggest that the glycosylation site in the folded TTR tetramer may not be accessible to glycosylating enzymes and/or their substrates. However, some variant TTR tetramers (e.g., L12P, and to a lesser extent V30M) have altered folded structures that expose Asn 98 and do get glycosylated [75]. Dissociation of tetramer to monomer (or partially unfolded, or unfolded monomer) also causes structural changes that can make the sequon accessible for glycosylation. If glycosylation is a factor in tagging TTR monomers for rapid proteolytic degradation, decreased glycosylation activity could result in a sufficient buildup of monomer to cause development of amyloidosis. The current data are insufficient to propose decreased rates of glycosylation or other posttranslational modification as the causative factor in triggering amyloid formation, but this scenario does emphasize that many additional plausible examples of types of reactions must be considered in seeking the causative factor initiating TTR amyloidosis. We hope that this model and the proposed quantitative metrics to identify amyloidosis early, will enable investigation of modified metabolic activities like glycosylation, as well as a method to quantify treatment efficacy which will enable development of a real cure for these diseases.

### Summary

1. This biophysical model and proposed measurement methods can be used directly with current human subjects or with banked blood samples. We propose that it will be of great benefit in current clinical trials to understand treatment efficacy.

2. The rate of synthesis of TTR is constant. For proteostasis, the rate of removal of TTR must equal the rate of synthesis. TTR in plasma is largely in the tetrameric form, (TTR)_4_, but dissociates to give very low, but significant concentrations of dimers and monomers.

3. Removal of TTR from plasma proceeds via monomers.

4. Monomers undergo two processes that remove them from solution, proteolysis or aggregation. The combined rates of these two pathways equals the total rate of monomer removal, which is also equal to the rate of production of monomer via dissociation of tetramer. Depending on the relative rates, either of the two reaction pathways could account for anywhere from 100% to 0% of the rate of monomer removal.

5. The critical monomer concentration for aggregation is unknown, however the cause of aggregation develops slowly over time. Once amyloidosis begins, the rate of development of amyloidosis is determined by the rate of monomer incorporation into various aggregates that lead to fibrils and amyloids.

6. Destabilizing tetramer by pleiotropic mutations leads to greater dissociation of monomer and a higher, variant-dependent concentration of TTR monomer in plasma. Mutations are not required for TTR amyloidosis formation; point mutations only modify the equilibrium concentrations in Figure 6. Amyloidosis caused by wild-type TTR follows the same mechanism as amyloidosis caused by variants of TTR and thus should be considered as variants of the same disease for purposes of clinical studies.

7. Amyloidosis begins when the rate of TTR proteolysis decreases relative to the rate of amyloid formation and monomer concentration increases sufficiently to allow significant oligomerization into fibrils and amyloids. The cause of a decrease in the rate of proteolysis of TTR remains to be identified.

When the tetramer is stabilized by drugs or stabilizing mutations, the concentration of tetramer will increase in plasma to a steady-state level determined by the rate of proteolysis.

## MATERIALS AND METHODS

### Chemical denaturation

Blood serum from a healthy human control was diluted with PBS to 10 mg/ml and divided into 9 fractions containing 0.02 milliliters. We added 0.027 milliliter guanidine of different stock solutions to each fraction to bring final guanidine concentration to 0, 0.4, 0.9, 1.3, 1.7, 2.2, 2.6, 3.0, 3.5M and partially unfold the proteins in the individual fractions as shown in Figure 4. This mixture was allowed to incubate at 37° C for 30 minutes at which point iodine/KI solution (0.3 molar) was added to achieve final concentration of 2.9 nmole in 0.05 milliliter. The iodine-labeling reaction proceeded for 10 minutes until 100mM imidazole was added to quench the reaction. At this point high concentration guanidine (6M) was added to fully denature all protein. Tris(2-carboxyethyl)phosphine (TCEP, final conc 10mM) was added to reduce disulfide linkages and chloroacetamide (40mM final concentration) was added to cap the reduced cysteines. The mixture was placed on top of a 30kD spin concentrator (VWR) and washed 2 times with ammonium bicarbonate (25 mM, ph=8.5). Trypsin (mass spectrometry grade) was added to the mixture on top of the filter and allowed to digest the protein overnight at 37° C. After digestion, peptides were spun through the filter and concentrated under vacuum for analysis by mass spectrometry.

### Thermal denaturation

Blood serum was diluted with PBS to 2 mg/ml and 30uL volume was added to 10 PCR tubes. A PCR thermocycler (Biorad T100) was used to create an equally spaced gradient of temperature with 10 steps from 37 to 63 or 37 to 100 Celsius. Samples were incubated at these temperatures for 3 minutes to partially unfold the proteins in the individual fractions, then cool down to 4° C for 3min to quench the reaction as shown in Figure 4. The samples were then removed from the thermocycler and spin at 14000xG at 4° C for 20mins to precipitate the aggregated protein. The soluble fraction was collected for protein digestion. At this point high concentration guanidine (6M) was added to fully denature protein. Tris(2-carboxyethyl)phosphine (TCEP, final conc 10mM) was added to reduce disulfide linkages and chloroacetamide (40mM final concentration) was added to cap the reduced cysteines. The mixture was placed on top of a 30kD spin concentrator (VWR) and washed 2 times with ammonium bicarbonate (25 mM, ph=8.5). Trypsin (mass spectrometry grade) was added to the mixture on top of the filter and allowed to digest the protein overnight at 37° C. After digestion peptides were spun through the filter and concentrated under vacuum for analysis by mass spectrometry.

## References

[1] Adams D, Koike H, Slama M, Coelho T. Hereditary transthyretin amyloidosis: a model of medical progress for a fatal disease. Nat Rev Neurol. 2019; 15:387–404. 10.1038/s41582-019-0210-4 31209302

[2] Ruberg FL, Grogan M, Hanna M, Kelly JW, Maurer MS. Transthyretin amyloid cardiomyopathy: JACC state-of-the-art review. J Am Coll Cardiol. 2019; 73:2872–91. 10.1016/j.jacc.2019.04.003 31171094 PMC6724183

[3] Sekijima Y, Wiseman RL, Matteson J, Hammarström P, Miller SR, Sawkar AR, Balch WE, Kelly JW. The biological and chemical basis for tissue-selective amyloid disease. Cell. 2005; 121:73–85. 10.1016/j.cell.2005.01.018 15820680

[4] Swiecicki PL, Zhen DB, Mauermann ML, Kyle RA, Zeldenrust SR, Grogan M, Dispenzieri A, Gertz MA. Hereditary ATTR amyloidosis: a single-institution experience with 266 patients. Amyloid. 2015; 22:123–31. 10.3109/13506129.2015.1019610 26017327

[5] Westermark GT, Fändrich M, Westermark P. AA amyloidosis: pathogenesis and targeted therapy. Annu Rev Pathol. 2015; 10:321–44. 10.1146/annurev-pathol-020712-163913 25387054

[6] Herbert J, Wilcox JN, Pham KT, Fremeau RT Jr, Zeviani M, Dwork A, Soprano DR, Makover A, Goodman DS, Zimmerman EA. Transthyretin: a choroid plexus-specific transport protein in human brain. The 1986 S. Weir mitchell award. Neurology. 1986; 36:900–11. 10.1212/wnl.36.7.900 3714052

[7] Ashe KH, Aguzzi A. Prions, prionoids and pathogenic proteins in Alzheimer disease. Prion. 2013; 7:55–59. 10.4161/pri.23061 23208281 PMC3609051

[8] Leach BI, Zhang X, Kelly JW, Dyson HJ, Wright PE. NMR measurements reveal the structural basis of transthyretin destabilization by pathogenic mutations. Biochemistry. 2018; 57:4421–30. 10.1021/acs.biochem.8b00642 29972637 PMC6067956

[9] Saraiva MJ. Transthyretin amyloidosis: a tale of weak interactions. FEBS Lett. 2001; 498:201–03. 10.1016/s0014-5793(01)02480-2 11412857

[10] Soto C, Estrada L, Castilla J. Amyloids, prions and the inherent infectious nature of misfolded protein aggregates. Trends Biochem Sci. 2006; 31:150–55. 10.1016/j.tibs.2006.01.002 16473510

[11] Lemarre P, Pujo-Menjouet L, Sindi SS. Generalizing a mathematical model of prion aggregation allows strain coexistence and co-stability by including a novel misfolded species. J Math Biol. 2019; 78:465–95. 10.1007/s00285-018-1280-4 30116882 PMC6399074

[12] Lin MS, Chen LY, Tsai HT, Wang SS, Chang Y, Higuchi A, Chen WY. Investigation of the mechanism of beta-amyloid fibril formation by kinetic and thermodynamic analyses. Langmuir. 2008; 24:5802–08. 10.1021/la703369b 18452319

[13] Verma M, Girdhar A, Patel B, Ganguly NK, Kukreti R, Taneja V. Q-rich yeast prion [*PSI*^+^] accelerates aggregation of transthyretin, a non-Q-rich human protein. Front Mol Neurosci. 2018; 11:75. 10.3389/fnmol.2018.00075 29593496 PMC5859028

[14] Saelices L, Chung K, Lee JH, Cohn W, Whitelegge JP, Benson MD, Eisenberg DS. Amyloid seeding of transthyretin by ex vivo cardiac fibrils and its inhibition. Proc Natl Acad Sci U S A. 2018; 115:E6741–E50. 29954863 10.1073/pnas.1805131115 PMC6055172

[15] Lohmann S, Bernis ME, Tachu BJ, Ziemski A, Grigoletto J, Tamgüney G. Oral and intravenous transmission of α-synuclein fibrils to mice. Acta Neuropathol. 2019; 138:515–33. 10.1007/s00401-019-02037-5 31230104 PMC6778172

[16] Makover A, Moriwaki H, Ramakrishnan R, Saraiva MJ, Blaner WS, Goodman DS. Plasma transthyretin. Tissue sites of degradation and turnover in the rat. J Biol Chem. 1988; 263:8598–603. 3379035

[17] Connelly S, Mortenson DE, Choi S, Wilson IA, Powers ET, Kelly JW, Johnson SM. Semi-quantitative models for identifying potent and selective transthyretin amyloidogenesis inhibitors. Bioorg Med Chem Lett. 2017; 27:3441–49. 10.1016/j.bmcl.2017.05.080 28625364 PMC5557047

[18] Wiseman RL, Johnson SM, Kelker MS, Foss T, Wilson IA, Kelly JW. Kinetic stabilization of an oligomeric protein by a single ligand binding event. J Am Chem Soc. 2005; 127:5540–51. 10.1021/ja042929f 15826192

[19] Berk J, Connors L, Hankinson E, Falzone R, Figueroa Y, Hutabarat R, Butler J, Kretschmer M, Sah DWY, Cehelsky J, Vaishnaw A, Gollob J. Serial Measurements of Circulating Transthyretin (Ttr) in Subjects with Ttr Amyloidosis or Carriers of Mutant Ttr. Journal of the Peripheral Nervous System. 2011; 16:S10–S1.

[20] Judge DP, Heitner SB, Falk RH, Maurer MS, Shah SJ, Witteles RM, Grogan M, Selby VN, Jacoby D, Hanna M, Nativi-Nicolau J, Patel J, Rao S, et al. Transthyretin stabilization by AG10 in symptomatic transthyretin amyloid cardiomyopathy. J Am Coll Cardiol. 2019; 74:285–95. 10.1016/j.jacc.2019.03.012 30885685

[21] Morimoto RI. Cell-nonautonomous regulation of proteostasis in aging and disease. Cold Spring Harb Perspect Biol. 2020; 12:a034074. 10.1101/cshperspect.a034074 30962274 PMC7111247

[22] Santra M, Dill KA, de Graff AM. Proteostasis collapse is a driver of cell aging and death. Proc Natl Acad Sci USA. 2019; 116:22173–78. 10.1073/pnas.1906592116 31619571 PMC6825304

[23] Cuervo AM, Dice JF. How do intracellular proteolytic systems change with age? Front Biosci. 1998; 3:d25–43. 10.2741/a264 9407152

[24] Gomes R, Sousa Silva M, Quintas A, Cordeiro C, Freire A, Pereira P, Martins A, Monteiro E, Barroso E, Ponces Freire A. Argpyrimidine, a methylglyoxal-derived advanced glycation end-product in familial amyloidotic polyneuropathy. Biochem J. 2005; 385:339–45. 10.1042/BJ20040833 15281912 PMC1134703

[25] Bulawa CE, Connelly S, Devit M, Wang L, Weigel C, Fleming JA, Packman J, Powers ET, Wiseman RL, Foss TR, Wilson IA, Kelly JW, Labaudinière R. Tafamidis, a potent and selective transthyretin kinetic stabilizer that inhibits the amyloid cascade. Proc Natl Acad Sci USA. 2012; 109:9629–34. 10.1073/pnas.1121005109 22645360 PMC3386102

[26] Falk RH. Senile systemic amyloidosis: are regional differences real or do they reflect different diagnostic suspicion and use of techniques? Amyloid. 2012 (Suppl 1); 19:68–70. 10.3109/13506129.2012.674074 22620969

[27] Gertz MA, Benson MD, Dyck PJ, Grogan M, Coelho T, Cruz M, Berk JL, Plante-Bordeneuve V, Schmidt HH, Merlini G. Diagnosis, prognosis, and therapy of transthyretin amyloidosis. J Am Coll Cardiol. 2015; 66:2451–66. 10.1016/j.jacc.2015.09.075 26610878

[28] Holmgren G, Wikström L, Lundgren HE, Suhr OB. Discordant penetrance of the trait for familial amyloidotic polyneuropathy in two pairs of monozygotic twins. J Intern Med. 2004; 256:453–56. 10.1111/j.1365-2796.2004.01399.x 15485482

[29] Judge DP, Lee YZ, Sharma K. Untangling wild-type transthyretin amyloidosis. J Am Coll Cardiol. 2016; 68:1021–23. 10.1016/j.jacc.2016.06.032 27585506

[30] Hammarström P, Jiang X, Hurshman AR, Powers ET, Kelly JW. Sequence-dependent denaturation energetics: a major determinant in amyloid disease diversity. Proc Natl Acad Sci USA. 2002 (Suppl 4); 99:16427–32. 10.1073/pnas.202495199 12351683 PMC139904

[31] Sebastião MP, Lamzin V, Saraiva MJ, Damas AM. Transthyretin stability as a key factor in amyloidogenesis: x-ray analysis at atomic resolution. J Mol Biol. 2001; 306:733–44. 10.1006/jmbi.2000.4415 11243784

[32] Maurer MS, Bokhari S, Damy T, Dorbala S, Drachman BM, Fontana M, Grogan M, Kristen AV, Lousada I, Nativi-Nicolau J, Cristina Quarta C, Rapezzi C, Ruberg FL, et al. Expert consensus recommendations for the suspicion and diagnosis of transthyretin cardiac amyloidosis. Circ Heart Fail. 2019; 12:e006075. 10.1161/CIRCHEARTFAILURE.119.006075 31480867 PMC6736650

[33] Foss TR, Wiseman RL, Kelly JW. The pathway by which the tetrameric protein transthyretin dissociates. Biochemistry. 2005; 44:15525–33. 10.1021/bi051608t 16300401

[34] Johnson SM, Wiseman RL, Sekijima Y, Green NS, Adamski-Werner SL, Kelly JW. Native state kinetic stabilization as a strategy to ameliorate protein misfolding diseases: a focus on the transthyretin amyloidoses. Acc Chem Res. 2005; 38:911–21. 10.1021/ar020073i 16359163

[35] Quintas A, Vaz DC, Cardoso I, Saraiva MJ, Brito RM. Tetramer dissociation and monomer partial unfolding precedes protofibril formation in amyloidogenic transthyretin variants. J Biol Chem. 2001; 276:27207–13. 10.1074/jbc.M101024200 11306576

[36] Purkey HE, Dorrell MI, Kelly JW. Evaluating the binding selectivity of transthyretin amyloid fibril inhibitors in blood plasma. Proc Natl Acad Sci USA. 2001; 98:5566–71. 10.1073/pnas.091431798 11344299 PMC33253

[37] Schmidt M, Wiese S, Adak V, Engler J, Agarwal S, Fritz G, Westermark P, Zacharias M, Fändrich M. cryo-EM structure of a transthyretin-derived amyloid fibril from a patient with hereditary ATTR amyloidosis. Nat Commun. 2019; 10:5008. 10.1038/s41467-019-13038-z 31676763 PMC6825171

[38] Chaudhuri TK, Paul S. Protein-misfolding diseases and chaperone-based therapeutic approaches. FEBS J. 2006; 273:1331–49. 10.1111/j.1742-4658.2006.05181.x 16689923

[39] Privalov PL, Dragan AI. Microcalorimetry of biological macromolecules. Biophys Chem. 2007; 126:16–24. 10.1016/j.bpc.2006.05.004 16781052

[40] Privalov PL, Khechinashvili NN. A thermodynamic approach to the problem of stabilization of globular protein structure: a calorimetric study. J Mol Biol. 1974; 86:665–84. 10.1016/0022-2836(74)90188-0 4368360

[41] Shi J, Hu Y, Smith DE, Zhu HJ. A sensitive liquid chromatography-tandem mass spectrometry method for the quantification of valacyclovir and its metabolite acyclovir in mouse and human plasma. J Chromatogr B Analyt Technol Biomed Life Sci. 2018; 1092:447–52. 10.1016/j.jchromb.2018.06.040 29945109 PMC6882412

[42] Mateus A, Kurzawa N, Becher I, Sridharan S, Helm D, Stein F, Typas A, Savitski MM. Thermal proteome profiling for interrogating protein interactions. Mol Syst Biol. 2020; 16:e9232. 10.15252/msb.20199232 32133759 PMC7057112

[43] Walker EJ, Bettinger JQ, Welle KA, Hryhorenko JR, Ghaemmaghami S. Global analysis of methionine oxidation provides a census of folding stabilities for the human proteome. Proc Natl Acad Sci USA. 2019; 116:6081–90. 10.1073/pnas.1819851116 30846556 PMC6442572

[44] Bakalarski CE, Kirkpatrick DS. A biologist’s field guide to multiplexed quantitative proteomics. Mol Cell Proteomics. 2016; 15:1489–97. 10.1074/mcp.O115.056986 26873251 PMC4858934

[45] Lindemann C, Thomanek N, Hundt F, Lerari T, Meyer HE, Wolters D, Marcus K. Strategies in relative and absolute quantitative mass spectrometry based proteomics. Biol Chem. 2017; 398:687–99. 10.1515/hsz-2017-0104 28282288

[46] Vidova V, Spacil Z. A review on mass spectrometry-based quantitative proteomics: targeted and data independent acquisition. Anal Chim Acta. 2017; 964:7–23. 10.1016/j.aca.2017.01.059 28351641

[47] Zhang Y, Fonslow BR, Shan B, Baek MC, Yates JR 3rd. Protein analysis by shotgun/bottom-up proteomics. Chem Rev. 2013; 113:2343–94. 10.1021/cr3003533 23438204 PMC3751594

[48] Donato LJ, Lueke A, Kenyon SM, Meeusen JW, Camilleri M. Description of analytical method and clinical utility of measuring serum 7-alpha-hydroxy-4-cholesten-3-one (7aC4) by mass spectrometry. Clin Biochem. 2018; 52:106–11. 10.1016/j.clinbiochem.2017.10.008 29051033

[49] Booth DR, Sunde M, Bellotti V, Robinson CV, Hutchinson WL, Fraser PE, Hawkins PN, Dobson CM, Radford SE, Blake CC, Pepys MB. Instability, unfolding and aggregation of human lysozyme variants underlying amyloid fibrillogenesis. Nature. 1997; 385:787–93. 10.1038/385787a0 9039909

[50] Ferreon AC, Bolen DW. Thermodynamics of denaturant-induced unfolding of a protein that exhibits variable two-state denaturation. Biochemistry. 2004; 43:13357–69. 10.1021/bi048666j 15491142

[51] Hamuro Y, Coales SJ, Southern MR, Nemeth-Cawley JF, Stranz DD, Griffin PR. Rapid analysis of protein structure and dynamics by hydrogen/deuterium exchange mass spectrometry. J Biomol Tech. 2003; 14:171–82. 13678147 PMC2279949

[52] Leuenberger P, Ganscha S, Kahraman A, Cappelletti V, Boersema PJ, von Mering C, Claassen M, Picotti P. Cell-wide analysis of protein thermal unfolding reveals determinants of thermostability. Science. 2017; 355:eaai7825. 10.1126/science.aai7825 28232526

[53] Schopper S, Kahraman A, Leuenberger P, Feng Y, Piazza I, Müller O, Boersema PJ, Picotti P. Measuring protein structural changes on a proteome-wide scale using limited proteolysis-coupled mass spectrometry. Nat Protoc. 2017; 12:2391–410. 10.1038/nprot.2017.100 29072706

[54] Strickland EC, Geer MA, Tran DT, Adhikari J, West GM, DeArmond PD, Xu Y, Fitzgerald MC. Thermodynamic analysis of protein-ligand binding interactions in complex biological mixtures using the stability of proteins from rates of oxidation. Nat Protoc. 2013; 8:148–61. 10.1038/nprot.2012.146 23257983 PMC3717606

[55] Liu D, Ren D, Huang H, Dankberg J, Rosenfeld R, Cocco MJ, Li L, Brems DN, Remmele RL Jr. Structure and stability changes of human IgG1 fc as a consequence of methionine oxidation. Biochemistry. 2008; 47:5088–100. 10.1021/bi702238b 18407665

[56] Baslé E, Joubert N, Pucheault M. Protein chemical modification on endogenous amino acids. Chem Biol. 2010; 17:213–27. 10.1016/j.chembiol.2010.02.008 20338513

[57] Pal S, Pyne P, Samanta N, Ebbinghaus S, Mitra RK. Thermal stability modulation of the native and chemically-unfolded state of bovine serum albumin by amino acids. Phys Chem Chem Phys. 2019; 22:179–88. 10.1039/c9cp04887a 31799558

[58] Planté-Bordeneuve V, Said G. Familial amyloid polyneuropathy. Lancet Neurol. 2011; 10:1086–97. 10.1016/S1474-4422(11)70246-0 22094129

[59] de la Chapelle A, Tolvanen R, Boysen G, Santavy J, Bleeker-Wagemakers L, Maury CP, Kere J. Gelsolin-derived familial amyloidosis caused by asparagine or tyrosine substitution for aspartic acid at residue 187. Nat Genet. 1992; 2:157–60. 10.1038/ng1092-157 1338910

[60] Pepys MB, Hawkins PN, Booth DR, Vigushin DM, Tennent GA, Soutar AK, Totty N, Nguyen O, Blake CC, Terry CJ. Human lysozyme gene mutations cause hereditary systemic amyloidosis. Nature. 1993; 362:553–57. 10.1038/362553a0 8464497

[61] Benson MD, Liepnieks J, Uemichi T, Wheeler G, Correa R. Hereditary renal amyloidosis associated with a mutant fibrinogen alpha-chain. Nat Genet. 1993; 3:252–55. 10.1038/ng0393-252 8097946

[62] Yu L, Edalji R, Harlan JE, Holzman TF, Lopez AP, Labkovsky B, Hillen H, Barghorn S, Ebert U, Richardson PL, Miesbauer L, Solomon L, Bartley D, et al. Structural characterization of a soluble amyloid beta-peptide oligomer. Biochemistry. 2009; 48:1870–77. 10.1021/bi802046n 19216516

[63] David DC, Ollikainen N, Trinidad JC, Cary MP, Burlingame AL, Kenyon C. Widespread protein aggregation as an inherent part of aging in C. Elegans. PLoS Biol. 2010; 8:e1000450. 10.1371/journal.pbio.1000450 20711477 PMC2919420

[64] Moehle EA, Shen K, Dillin A. Mitochondrial proteostasis in the context of cellular and organismal health and aging. J Biol Chem. 2019; 294:5396–407. 10.1074/jbc.TM117.000893 29622680 PMC6462515

[65] Mathis AD, Naylor BC, Carson RH, Evans E, Harwell J, Knecht J, Hexem E, Peelor FF 3rd, Miller BF, Hamilton KL, Transtrum MK, Bikman BT, Price JC. Mechanisms of in vivo ribosome maintenance change in response to nutrient signals. Mol Cell Proteomics. 2017; 16:243–54. 10.1074/mcp.M116.063255 27932527 PMC5294211

[66] Dai DF, Karunadharma PP, Chiao YA, Basisty N, Crispin D, Hsieh EJ, Chen T, Gu H, Djukovic D, Raftery D, Beyer RP, MacCoss MJ, Rabinovitch PS. Altered proteome turnover and remodeling by short-term caloric restriction or rapamycin rejuvenate the aging heart. Aging Cell. 2014; 13:529–39. 10.1111/acel.12203 24612461 PMC4040127

[67] Karunadharma PP, Basisty N, Dai DF, Chiao YA, Quarles EK, Hsieh EJ, Crispin D, Bielas JH, Ericson NG, Beyer RP, MacKay VL, MacCoss MJ, Rabinovitch PS. Subacute calorie restriction and rapamycin discordantly alter mouse liver proteome homeostasis and reverse aging effects. Aging Cell. 2015; 14:547–57. 10.1111/acel.12317 25807975 PMC4531069

[68] Price JC, Khambatta CF, Li KW, Bruss MD, Shankaran M, Dalidd M, Floreani NA, Roberts LS, Turner SM, Holmes WE, Hellerstein MK. The effect of long term calorie restriction on in vivo hepatic proteostatis: a novel combination of dynamic and quantitative proteomics. Mol Cell Proteomics. 2012; 11:1801–14. 10.1074/mcp.M112.021204 22984287 PMC3518108

[69] Li L, Sawashita J, Ding X, Yang M, Xu Z, Miyahara H, Mori M, Higuchi K. Caloric restriction reduces the systemic progression of mouse AApoAII amyloidosis. PLoS One. 2017; 12:e0172402. 10.1371/journal.pone.0172402 28225824 PMC5321440

[70] Mouton PR, Chachich ME, Quigley C, Spangler E, Ingram DK. Caloric restriction attenuates amyloid deposition in middle-aged dtg APP/PS1 mice. Neurosci Lett. 2009; 464:184–87. 10.1016/j.neulet.2009.08.038 19699265 PMC2748166

[71] Sawashita J, Li L, Liu Y, Ding X, Yang M, Xu Z, Higuchi K. Caloric restriction prevents the progression of murine AApoAII amyloidosis. Amyloid. 2017; 24:171–72. 10.1080/13506129.2017.1295948 28434314

[72] Lee HS, Qi Y, Im W. Effects of n-glycosylation on protein conformation and dynamics: protein data bank analysis and molecular dynamics simulation study. Sci Rep. 2015; 5:8926. 10.1038/srep08926 25748215 PMC4352867

[73] Ruhaak LR, Uh HW, Beekman M, Hokke CH, Westendorp RG, Houwing-Duistermaat J, Wuhrer M, Deelder AM, Slagboom PE. Plasma protein N-glycan profiles are associated with calendar age, familial longevity and health. J Proteome Res. 2011; 10:1667–74. 10.1021/pr1009959 21184610

[74] Teixeira AC, Saraiva MJ. Presence of N-glycosylated transthyretin in plasma of V30M carriers in familial amyloidotic polyneuropathy: an escape from ERAD. J Cell Mol Med. 2013; 17:429–35. 10.1111/jcmm.12024 23387326 PMC3823024

[75] Sato T, Sako Y, Sho M, Momohara M, Suico MA, Shuto T, Nishitoh H, Okiyoneda T, Kokame K, Kaneko M, Taura M, Miyata M, Chosa K, et al. STT3B-dependent posttranslational N-glycosylation as a surveillance system for secretory protein. Mol Cell. 2012; 47:99–110. 10.1016/j.molcel.2012.04.015 22607976

[76] Krištić J, Vučković F, Menni C, Klarić L, Keser T, Beceheli I, Pučić-Baković M, Novokmet M, Mangino M, Thaqi K, Rudan P, Novokmet N, Sarac J, et al. Glycans are a novel biomarker of chronological and biological ages. J Gerontol A Biol Sci Med Sci. 2014; 69:779–89. 10.1093/gerona/glt190 24325898 PMC4049143

